# Medication Adherence in Patients with Rheumatoid Arthritis: The Effect of Patient Education, Health Literacy, and Musculoskeletal Ultrasound

**DOI:** 10.1155/2015/150658

**Published:** 2015-04-28

**Authors:** Samantha Joplin, Rick van der Zwan, Fredrick Joshua, Peter K. K. Wong

**Affiliations:** ^1^School of Psychology, University of Sydney, Sydney, NSW 2006, Australia; ^2^Department of Psychology, Southern Cross University, Coffs Harbour, NSW 2450, Australia; ^3^Department of Rheumatology, Prince of Wales Hospital, Randwick, Sydney, NSW 2031, Australia; ^4^Prince of Wales Hospital Clinical School, University of New South Wales, Randwick, Sydney, NSW 2031, Australia; ^5^Mid-North Coast Arthritis Clinic, Coffs Harbour, NSW 2450, Australia; ^6^Rural Clinical School, University of New South Wales, Coffs Harbour, NSW 2450, Australia

## Abstract

*Background*. Rheumatoid arthritis (RA) is a chronic systemic inflammatory disease affecting <1% of the population. Incompletely controlled RA results in fatigue, joint and soft tissue pain, progressive joint damage, reduced quality of life, and increased cardiovascular mortality. Despite an increasing range of disease modifying agents which halt disease progression, poor patient adherence with medication is a significant barrier to management. *Objective*. The goal of this review was to examine the effectiveness of measures to improve patient medication adherence. *Methods*. Studies addressing treatment adherence in patients with RA were identified by trawling PsycINFO, Medline, Cochrane, Pubmed, and ProQuest for studies published between January 2000 and October 2014. Articles were independently reviewed to identify relevant studies. *Results*. Current strategies were of limited efficacy in improving patient adherence with medications used to treat RA. *Conclusion*. Poor medication adherence is a complex issue. Low educational levels and limited health literacy are contributory factors. Psychological models may assist in explaining medication nonadherence. Increasing patient knowledge of their disease seems sensible. Existing educational interventions appear ineffective at improving medication adherence, probably due to an overemphasis on provision of biomedical information. A novel approach to patient education using musculoskeletal ultrasound is proposed.

## 1. Introduction

Rheumatoid arthritis (RA) is a chronic systemic autoimmune inflammatory disease with a prevalence of approximately 1% [1, 2]. Incompletely controlled RA results in severe progressive joint damage, functional disability, morbidity, and increased mortality [3]. Clarification of the molecular pathogenesis of RA has led to an increasing number of targeted therapies [4, 5]. Early intervention with disease-modifying antirheumatic drugs (DMARDs) and biological DMARDs (bDMARDs) improves long-term functional outcomes [6–9]. Depending upon the clinical situation, a realistic goal for every patient with RA is now low disease activity or disease remission [10].

Despite extensive evidence regarding drug efficacy and the risk of long-term harm from uncontrolled RA, medication adherence rates remain suboptimal, ranging from 30 to 80% [11–13]. Improving medication adherence with currently available DMARDs and bDMARDs would improve treatment effectiveness and reduce healthcare costs [14, 15]. Medication nonadherence is a dynamic, multifaceted issue affected by (i) patient factors, (ii) disease features, and (iii) drug characteristics. Although more effective interventions for improving medication adherence are needed [16], medication nonadherence remains a poorly studied phenomenon [17].

Medication nonadherence can be classified as primary or secondary, both of which are influenced by different factors [18]. Primary nonadherence occurs when a patient fails to fill an initial prescription [19]. This is often influenced by socioeconomic factors, such as out-of-pocket medication costs [18, 20]. Secondary nonadherence occurs when a patient prematurely discontinues the medication [19]. This may be associated with factors such as lack of drug efficacy, slow disease response to treatment, and adverse drug reactions.

Both primary and secondary medication nonadherence may be affected by low levels of health literacy and patient education. These factors may compromise patient understanding of the adverse outcomes from poorly controlled RA. Patients with RA often lack sufficient understanding to make informed health-care decisions [21]. In contrast, those with greater appreciation of the risks and benefits of treatment were more likely to accept risk to achieve better outcomes [21]. Unfortunately, patient educational interventions usually have limited effectiveness at improving medication adherence [3, 12, 22–24].

While many factors influence drug adherence, this review will focus on the effectiveness of existing educational and health literacy interventions targeting medication adherence in patients with RA. Psychological models of medication adherence and, within this context, current and past educational interventions will be explored. The effect of health literacy and the utility of musculoskeletal ultrasound (MSKUS) as an educational tool will also be discussed.

## 2. Methods

We performed a computerised systematic literature search of five databases (PsycINFO, Medline, Cochrane, Pubmed and Proquest) using a wide range of search terms to identify English language, peer reviewed papers dealing with medication adherence in patients with RA published between January 2000 and October 2014 (Table 1). A similar search strategy was employed to identify relevant publications dealing with the effect of patient education and health literacy on medication adherence in patients with RA (Table 2). Citation tracking was also used to identify additional papers. The most relevant articles for inclusion in this review were identified by SJ, RVDZ, and PKKW using strict inclusion and exclusion criteria (Table 3).

This review favours the term “adherence” over “compliance” as it reflects a shift in the clinician-patient relationship since the 1950s when the term “compliance” was commonly used [25]. “Compliance” may have authoritarian connotations as it refers to concordance of patient behaviour with medical advice [11, 26]. In contrast, “adherence” implies patient-clinician collaboration rather than obedience to didactic dissemination of medical advice.

## 3. Cognitive Models of Medication Adherence

Many factors affect medication adherence in patients with RA. In patients commencing the “anchor” DMARD methotrexate (MTX), adherence could be explained by a strong belief in the necessity of treatment, although it was not influenced by the severity of functional impairment [27]. Higher levels of medication adherence have been associated with participation in a patient education programme [28], following provision of more information about RA treatment [29], Caucasian ethnicity [30], and less disability [31]. Older age has been associated with both increased [27, 29, 32] and decreased [31, 33] medication adherence. Concomitant medication use has been associated with both higher [34] and lower [30] levels of medication adherence. Patients with stronger beliefs about the necessity of medication and who believed medications were generally not overused were more likely to be adherent to medication [34]. Rheumatoid disease activity had a variable relationship with medication adherence [31, 32]. Higher out-of-pocket costs [20], employment [27, 29], and cognitive impairment [29] were associated with reduced adherence rates.

As outlined above, the inability to consistently identify factors accurately predicting medication nonadherence in patients with RA has spurred development of cognitive models to better explain this complex phenomenon [11, 12]. One of the most widely used models is the Health Belief Model (HBM) which is a theoretical framework postulating that adherence decisions are based on implicit cost-benefit analyses, where the extent to which a patient views the necessity of medication is evaluated against concern about potential side effects [35]. The HBM was built on the premise that the likelihood of a person actively responding to a health threat depended on four key factors: (i) perceived illness threat (determined by disease severity and susceptibility), (ii) expectation of positive outcome (anticipated benefits of treatment), (iii) barriers associated with treatment (expected costs and drawbacks), and (iv) the extent to which they intend to adhere to treatment [36, 37].

The cost-benefit assessment offered by the HBM has been quantified as a necessity-concerns differential within the Beliefs about Medications Questionnaire (BMQ) [35, 38]. This user-friendly tool comprises two five-item scales evaluating patient beliefs about the necessity of medication relative to concern about adverse effects and predicts medication adherence more robustly than clinical or sociodemographic factors [35, 36]. When applied to a population with chronic illness such as asthma, renal or cardiac failure, and cancer, it was found that patients whose concern about medication outweighed their belief about the necessity of medication were less adherent to pharmacologic treatment [35]. This may have been an adaptive strategy to minimise potential harm from side effects or may have reflected how strongly patients believed medications were essential; that is, those believing them to be less necessary were more prone to forgetting to take them. Interestingly, many of the perceived “costs” arose from erroneous beliefs, for example, concerns regarding drug dependence [35].

Patients with RA often lacked the understanding required to make informed cost-benefit analyses leading to overestimation of medication risks [21, 39]. Medication risks were often thought to be high relative to surgery possibly because surgical risks were more tangible, making patients more likely to accept surgery even when this was associated with fewer benefits [21]. This highlights the need for effective educational interventions as patients well informed about the risks and benefits of medication performed more biomedically oriented cost-benefit analyses [39]. Those with a greater understanding of the risks and benefits of treatment were more inclined to accept risk in the pursuit of successful disease outcomes [21]. Unwillingness to accept risk compounded by poor understanding of the benefit of conventional biomedical treatment may explain the large number of patients using complementary or alternative medicines (CAMs) as these are often thought to have minimal risk [27, 40, 41]. While most CAMs are tested for safety, rigorous tests of efficacy are scarce [41, 42]. This highlights the importance of explaining the benefits and not just potential side effects of conventional treatment.

Clinicians should view patients as active decision-makers with a vested interest in their health who would be more adherent to medication if they believed the necessity of medication outweighed concerns about adverse effects [35]. Alas, due to medicolegal considerations clinicians often spend more time discussing the adverse effects of treatment rather than the benefits. This has probably been influenced by landmark cases such as the 1992 decision in* Rogers v. Whitaker *(1992) 175 CLR 479, which established in Australia the standard of care required when a doctor provides information to a patient about the risk of a proposed intervention [43]. The High Court of Australia affirmed that an ophthalmic surgeon should have warned his patient of the one in 14,000 chance of a rare complication (sympathetic ophthalmia) with its associated risk of blindness arising from a procedure. This was despite evidence tendered during the hearing that many of the defendant's colleagues would not have told their patients about the risk of such a rare complication.

The BMQ has been used in a cross-sectional study to describe the tension experienced by RA patients when assessing the importance of medication versus their concern regarding side effects [36]. Most respondents of a postal survey (*n* = 344) mailed to over 600 patients with RA agreed that their medication was necessary for health. However, almost half were concerned about potential adverse effects and this was associated with nonadherence [36]. The observed similarity in disease knowledge between adherent and nonadherent patients raises doubts about the effectiveness of educational interventions which merely increase knowledge [36].

Another psychological model used to describe medication adherence is Leventhal and colleagues' Self-Regulatory Model (SRM) [44, 45]. This is a hierarchically organised model of illness adaptation based on three primary constructs: illness representations, coping responses, and appraisal of coping responses [46, 47]. Illness representations or “lay beliefs” are defined as complex schemas from personal and familial experiences influencing how patients perceive and cope with chronic illness [48]. Cognitive and emotional illness representations form the crux of the SRM as these representations are integrated into patients' preexisting lay belief schemas and help them understand symptoms while moderating coping responses [46] and thus medication adherence [44].

A qualitative study involving semistructured interviews of 30 women with RA found that a positive patient-healthcare practitioner relationship was an important factor in the decision making process [30]. Potential and perceived adverse effects were powerful factors associated with nonadherence or discontinuation of medication [30]. These findings highlighted the importance of clear, helpful patient-practitioner communication and the need for healthcare practitioners to balance patient concerns about adverse effects with the likely benefits of treatment adherence.

Exploring medication adherence through the conceptual frameworks offered by the HBM and SRM suggests some practical strategies to improve medication adherence. Clinicians should address patient concerns about adverse effects by highlighting the positive outcomes associated with treatment [44]. The lack of association between factual knowledge and medication adherence highlights the need for more effective educational interventions [39]. The SRM suggests clinicians need to consider existing lay belief schemas used by patients to evaluate and cope with medical advice [46].

## 4. Assessment of Adherence

Medication adherence has been assessed by Electronic Medication Event Monitoring (EMEM) [49], pharmacy records [20, 50], self-report measures (e.g., the Compliance-Questionnaire-Rheumatology (CQR)) [49, 51], and more objective but invasive measures such as serum drug assays [28, 49]. Although susceptible to recall and social desirability biases, self-report measures represent the most pragmatic method for assessing medication adherence [35, 44]. Self-report measures such as the CQR provide clinicians with valuable information regarding how closely patients follow medication advice and their beliefs regarding the necessity of treatment [28, 35, 49]. The CQR is a 19-item rheumatology-specific questionnaire assessing medication adherence which also identifies factors associated with poor adherence [49, 51]. This tool demonstrated strong predictive validity when tested against EMEM [49]. A study of 126 RA patients which used the CQR to measure adherence and the BMQ to assess medication beliefs at initiation of MTX therapy found adherence rates during the first year of treatment could be explained by medication beliefs such as the perceived necessity of MTX [27]. Interestingly, supplying the treating clinician with information regarding medication adherence patterns does not influence patients' medication beliefs, nor does it engender any changes in adherence [52].

## 5. Educational Interventions and Medication Adherence

There is broad consensus regarding the importance of educational interventions for patients suffering from chronic illness such as RA [22]. A cross-sectional study of 33 RA patients with disease duration less than one year and 69 with disease duration greater than 10 years found both groups desired more information about their condition [53]. Educational interventions have usually entailed provision of information to patients about the disease and possible treatments [3] on the premise that increased knowledge leads to positive attitudes and behaviours which may be associated with small reductions in pain and disability [54, 55]. Many clinicians therefore regard education as important for equipping patients with the tools and coping strategies to manage disease flares [3, 56]. However, the effectiveness of educational interventions at improving medication adherence is questionable [3, 22, 23, 48], with potentially few short-term benefits [24].

A randomised controlled trial of 100 patients with RA found that patient education was associated with increased medication adherence [28]. Participants with active RA were randomised to an experimental group which received seven 30-minute one-on-one sessions with a rheumatology nurse aimed at increasing self-efficacy or a control group which received standard treatment. At the end of six months those in the experimental group were more adherent to medication [28]. However, others have found that patients with recent onset active RA had high levels of medication adherence regardless of participation in an educational programme which involved group meetings with an instructor who addressed erroneous patient beliefs and provided information about RA medication, the importance of physical activity, and joint conservation [23]. This suggested that educational interventions may not be needed in patients with recent onset active RA.

Although not specifically dealing with RA patients, a randomised controlled trial involving a pharmacist-delivered telephone service to patients at home led to increased medication adherence, fewer reports of medication side effects, and more positive medication beliefs [57]. This intervention appeared to shift the patient cost-benefit analysis to favour treatment benefits, thereby increasing the proportion of adherent patients [57].

A recent study tested the effect of a motivational interviewing (MI) programme on medication adherence in RA patients using a group-based format [58]. In this study, 123 participants were randomised to either the control or intervention group; the latter received two pharmacist-delivered MI sessions. These sessions aimed to resolve barriers to adherence with a practical, problem solving approach. The trial did not demonstrate any significant change to patient beliefs or medication adherence, possibly due to a “Hawthorne effect” or suboptimal integrity of the intervention [58].

In a pilot study, patients were randomly allocated to either group-based counselling or individual counselling [59]. Adherence, as measured by pill counts, was higher in patients who were counselled in a group-based format (90%) compared to those counselled individually (69%). However, probably due to lack of power, there was no statistically significant difference between treatment groups.

A systematic review found that educational interventions in patients with RA appeared to have a positive impact upon short-term outcomes but long-term improvements in health status were not clearly evident [22]. A Cochrane review of 31 randomised controlled trials found that patient education was associated with short-term benefits on disability, patient global assessment, and psychological status [60]. However, no long-term benefits were identified. Although clinical trials have demonstrated that educational interventions enhanced patient knowledge and understanding of their disease, there is conflicting evidence regarding their effects on medication adherence [3, 61]. This suggests that education alone is insufficient to increase medication adherence as medication beliefs appear to be influenced by more than information [3, 35].

Existing educational interventions may be limited by an overemphasis on improving patient knowledge [48]. This creates a power imbalance in the therapeutic interaction as the rheumatologist assumes the patient has no prior knowledge of their disease and should fulfil a passive role in the exchange and that provision of information is sufficient. Such assumptions give little credence to lay beliefs. While often exhibiting internal consistency, lay beliefs are seldom congruent with biomedical concepts but can strongly influence patient response to advice and treatment proposed by their rheumatologist [48].

When interviewed before and after a consultation with their rheumatologist, patients with RA were more accepting of and more adherent to suggested treatment if the advice provided during the consultation aligned with their lay beliefs [48]. Likewise, those who received information during the consultation incongruent with their lay beliefs were more likely to reject the management offered [48]. These findings can be interpreted through the conceptual framework of the SRM, which highlights the importance of existing patient lay belief schemas [46]. It is important to recognise that patient and clinician disease perceptions may diverge. Patient views are influenced by subjective information such as pain levels, whereas clinicians' views are usually informed by biomedical factors such as swollen and tender joint counts [62]. In light of such findings, an interactive educational process emphasising the active role of both clinician and patient might enable a more harmonious interweaving of biomedical information into preexisting lay belief systems [48].

## 6. Health Literacy and Medication Adherence

Health literacy refers to the ability to acquire, comprehend, and pursue health information to guide health-related decisions [55, 63, 64]. Individuals with limited health literacy have poorer health outcomes due to poor self-management, limited health responsibility, and underutilisation of healthcare resources [55]. Up to 42% of patients with chronic musculoskeletal disease may have low health literacy [65] while up to one-third of patients incorrectly followed dosing instructions for common rheumatology drugs [66]. A cross-sectional study of 110 patients with RA found that poor health literacy was associated with functional impairment as measured by the Multidimensional Health Assessment Questionnaire (MDHAQ) [67]. Analysis of data from 6052 patients with RA enrolled in a prospective observational study found that health literacy was found to predict functional status more robustly than corticosteroid and biologic use, smoking history, and education [68]. This suggests that some of the observed benefit on medication adherence following educational interventions may be due to a positive effect on patient health literacy and raises the possibility that better outcomes could be achieved by improving health literacy rather than semantic disease knowledge [67].

The multidimensional causal model of Paasche-Orlow and Wolf has been used to explain the link between low health literacy and poor patient outcomes [69]. This model attributed poor patient outcomes from low health literacy to the challenges posed by (i) communicating with clinicians, (ii) accessing and consuming health services, and (iii) effective self-care. Effective self-care requires patients to possess the knowledge and capacity to understand and implement their medication regime [69]. Low health literacy impairs patient ability to comprehend medication labels and instructions and to recall medication names [64, 69]. Accordingly, educational interventions should aim to increase self-care and patient awareness of available healthcare resources and minimise the communication barrier between patients and clinicians.

A practical suggestion is that clinicians should use visual tools such as videos and pictorial aids to assist in meaningful delivery of key health messages [64]. The beneficial effect of visual images within the consultation is further enhanced when combined with meaningful written or verbal information and has been shown to enhance patient comprehension and, ultimately, medication adherence [70]. This further highlights the need for clinician-patient collaboration within the consultation and a multimodal approach for communicating patient information.

## 7. The Use of Musculoskeletal Ultrasonography as an Educational Tool

Increasing evidence suggests that health interventions are more effective when they contain visual elements and simple, comprehensible information in an accessible format [64, 71–74]. Incorporation of pictures into medication instructions improved patient understanding, recollection, and adherence to medication regimes [70]. A double blind randomised controlled trial of 111 patients with early inflammatory arthritis found that provision of visual feedback for patients in the form of charts depicting disease activity significantly increased patient adherence to DMARDs [75]. This was associated with less disease activity at 12 months. These findings suggest that incorporating visual feedback into clinical practice may have a positive effect on treatment adherence and, ultimately, disease management.

**Figure 1:**
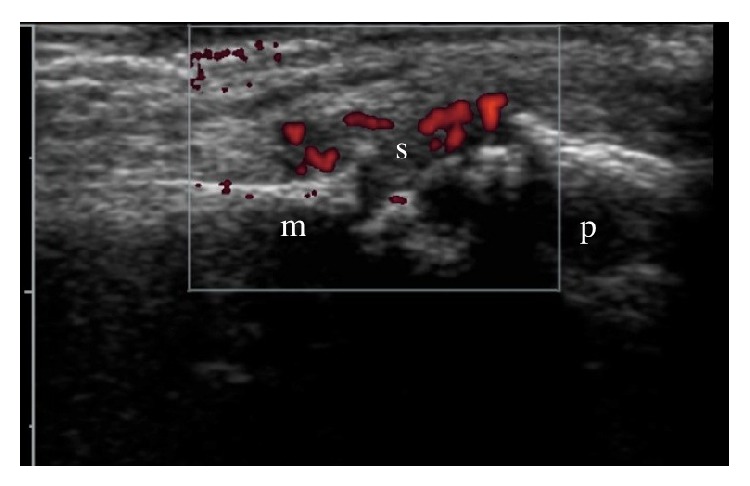
Longitudinal sonographic view of the right second metacarpophalangeal joint (m: metacarpal, p: proximal phalanx) in a patient with rheumatoid arthritis showing synovitis/effusion (s) with power Doppler flow (red).

Musculoskeletal ultrasound (MSKUS) is increasingly used to assist in diagnosis and monitoring of inflammatory arthritis and to guide joint injection and aspiration [76–79]. The presence of power Doppler signal (Figure 1) in patients whose RA appeared to be in clinical remission was helpful in predicting disease flare and radiographic outcome [80]. Auto-feedback from US assessment of joints in patients with RA quickly improved joint palpation skills [81]. A 12-week MSKUS course was useful in undergraduate teaching of joint anatomy and pathology [82]. Despite these benefits, MSKUS is underutilised in clinical practice and is routinely performed by less than 50% of rheumatologists in Europe [76]. Few rheumatologists in Australia routinely use MSKUS.

Musculoskeletal ultrasound may prove a valuable educational tool in clinical practice [83]. The treating clinician is able to enhance patient understanding “real-time” through demonstration of joint structures, articular and periarticular damage, and synovial inflammation. In particular, the ability to navigate around the site of interest and to show motion information may improve patient understanding more than provision of static images [84, 85]. Careful use of the device highlighting critical features for patients using their own anatomy is important in providing visual learning cues. This type of disease visualisation, when combined with clinician-patient interaction at close quarters, may improve adaptation to the diagnosis, thereby increasing medication adherence and disease self-control. A recent study involving patients with active RA (DAS28 score > 2.6) found that showing RA patients “real-time” US images of their clinically inflamed joints resulted in a more favourable cost-benefit analysis as measured by the BMQ, that is, increased patient belief in the necessity of medication versus concern about taking medication [86]. However, this finding needs to be confirmed by larger, longer-term studies.

## 8. Summary

The effectiveness of commonly used educational interventions targeting poor medication adherence is questionable. Educational interventions have generally focused on provision of information [48]. While these increase patient knowledge of the disease, they have not been reliably associated with increased medication adherence or improvement in long-term health status [24, 61]. There is a tension experienced by RA patients when assessing the necessity of medication against concern regarding adverse effects [36]. The goal of education should be to provide understandable information to patients to allow them to make informed healthcare decisions.

Educational interventions should incorporate more clinician-patient interaction [27]. Clinicians should deemphasise biomedical information and give more consideration to patient lay beliefs regarding clinical management without compromising use of evidence-based treatments that halt disease progression. This should allow clinicians to allay patient concerns while highlighting treatment benefits [35, 44]. Integrating MSKUS into an evidence-based educational framework may be beneficial because of its clinical value and as an educative tool to increase patient understanding of their disease [83, 86].

Musculoskeletal US may foster increased patient-clinician interaction and disease visualization, factors which have been lacking in traditional educational frameworks. Patients will also benefit from increased appreciation of joint structures and greater understanding of the long-term implications of joint damage and inflammation. These measures may address some of the limitations of previous interventions and hopefully result in increased patient medication adherence and better disease control.

## Figures and Tables

**Table 1:** Databases accessed displaying search terms employed, results, access limitation, reasons for exclusion and accepted papers dealing with factors affecting medication adherence in patients with rheumatoid arthritis.

Database *Search terms *	Number of papers identified	Number of full text peer reviewed papers able to be accessed	Reasons for exclusion (number)	Number of papers identified for inclusion [ref. number]
PsycINFO via EBSCO host *Rheumatoid arthritis * [and] *Compliance* [and] *Medication *	23	5	Juvenile population (1) Review paper (1) Not relevant (2)	*n* = 1 [34]

ProQuest (general) *Adherence* [And] *rheumatoid arthritis* [and] * Relationship *	3099	1891	Already included (1) Review papers (2) Juvenile population (1) Not relevant (1886)	*n* = 1 [87]

ProQuest (general) *Rheumatoid arthritis * [and] *Compliance* [and] *Medication *	4391	2118	Already included (1) Juvenile population (1) Not relevant (2114)	*n* = 2 [27, 28]

ProQuest (general) *Adherence *[or] c*ompliance * [and] *rheumatoid arthritis * [and] *Factors *[or] *predictors *	10265	5486	Already included (3) Not relevant (5481)	*n* = 2 [29, 32]

PubMed *rheumatoid arthritis* [and] *factors * [and] *Medication persistence *	48	15	Already included (3) Unrepresentative population (2) Not relevant (9)	*n* = 1 [31]

PubMed *Rheumatoid arthritis * [and] *Compliance * [and] *Medication *	163	138	Review/meta-analysis (2) Juvenile population (2) Not relevant (133)	*n* = 1 [20]

PubMed *Adherence* [or] *compliance * [and] *rheumatoid arthritis * [and] *Factors *[or] *predictors *	17	9	Osteoporosis management (1) Not relevant (7)	*n* = 1 [30]

**Table 2:** Databases accessed displaying search terms employed, quantity of results, access limitation, reasons for exclusion, and accepted papers dealing with the effect of patient education and literacy on medication adherence in patients with rheumatoid arthritis.

Database *Search terms *	Results	Reasons for exclusion	Accepted citations [ref. number]
**PsycINFO via OvidSP host** *Rheuma* ^*^ * arthritis [or] Rheuma* ^*^ * disease [or] RA* [and] *Complian* ^*^ * [or] non?compli* ^*^ * [or]* adherence *[or] non?adherence [or]* refusal *[or] regime* ^*^ [and] *Medic* ^*^ * [or]* pharmac^*^ [or] drug [or] treatment [or] therapy [or] biologic [or] ?DMARD? [or] disease?modifying^*^ [and] Patient education^*^ [or] intervention [or] strategy [or] knowledge [or] health liter^*^ [or] understanding	20	*n* = 2, juvenile/paediatric population *n* = 4, review/qualitative/ book *n* = 13, not relevant	*n* = 1 [58]

**EBM Reviews, All-Cochrane DSR, ACP Journal Club, DARE, and CCTR OvidSP host** *Rheuma* ^*^ * arthritis [or] RA [or] rheama* ^*^ * disease* [and] *Complian* ^*^ * [or] non?compli* ^*^ *[or]* adherence *[or] non?adherence [or]* refusal *[or] regime* [and] *Medic* ^*^ *[or]* pharmac^*^ [or] drug [or] treatment [or] therapy [or] biologic [or] ?DMARD?	473	*n* = 3, already included *n* = 469, not relevant	*n* = 1 [75]

**MedLine via OvidSP host** *Rheuma* ^*^ * arthritis [or] RA [or] rheama* ^*^ * disease* [and] *Complian* ^*^ *[or] non?compli* ^*^ *[or]* adherence *[or] non?adherence [or]* refusal *[or] regime* [and] *Medic* ^*^ * [or]* pharmac^*^ [or] drug [or] treatment [or] therapy [or] biologic [or] ?DMARD? [and] Medication	221	*n* = 3, already included *n* = 1, juvenile population *n* = 4, review/qualitative/ book *n* = 210, not relevant *n* = 1, female-only sample	*n* = 2 [28, 59]

The asterisk star is a wildcard search character. In this instance, it represents a string of characters, for example, the ∗ matches zero or more characters, so rheum ∗ will generate rheumatoid, rheumatology, rheumatic and so forth.

**Table 3:** Inclusion and exclusion criteria for identification of relevant papers.

Selection criteria	Inclusion criteria	Exclusion criteria
Participants	Human Adult American College of Rheumatology criteria for the diagnosis of RA	Animal Under 18 years Gender-specific Geriatric

Research design	Experimental Longitudinal	Qualitative Observational Case studies Editorials Cross-sectional Retrospective cohort study

Measurement scales	Validated questionnaires Blood assays Records/claims (pharmaceutical/insurance) Event monitoring	Interviews

RA-specific medication	Disease modifying antirheumatic drugs (DMARDS) Biological DMARDS Slow acting antirheumatic drugs (SAARDS)	Analgesics only Nonsteroidal anti-inflammatory drugs Corticosteroids only
